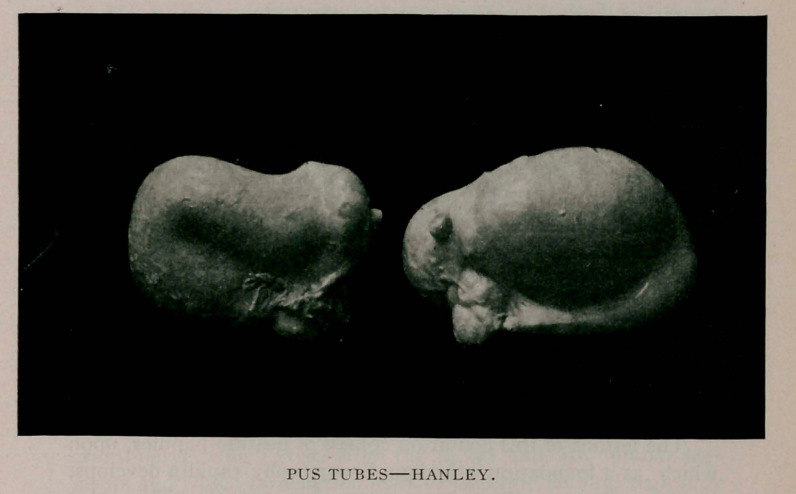# Removal of Two Large Pus Tubes

**Published:** 1902-01

**Authors:** L. G. Hanley, Charles Banta

**Affiliations:** Buffalo, N. Y., Gynecologist to the Buffalo Hospital of the Sisters of Charity; Interne


### CLINICAL REPORT.

### Removal of Two Large Pus Tubes.

By L. G. HANLEY, M. D., Buffalo, N. Y.,
Gynecologist to the Buffalo Hospital of the Sisters of Charity.
Reported by CHARLES BANTA, M. D., Interne.

Miss G., age 23; admitted to hospital, May 27, 1901. Age at first menstruation, 14. Family history good; father and mother living; sisters and brothers healthy. Has always been

PUS TUBES—HANLEY.

regular—menstrual period lasting four days,—no pain and in every respect normal; occurring every twenty-eight days until two years ago, when she received an injury while riding a wheel. She ran into a wagon, striking her abdomen against the vehicle. There has never been any history of leucorrhea or any discharge from vagina or dysmenorrhea prior to the injury or after it.

Menstruation ceased for ten weeks after the injury, when she had a severe attack of hemorrhage, accompanied by pain and convulsions.

The attacks of spasm and dyspnea would occur at various intervals, sometimes coming on every two weeks and lasting three or four days with four or five convulsions during twenty four hours. She had been informed she had an ovarian cyst and was referred to Dr. Hanley for operation. Patient was prepared for section and on opening the abdomen, examination revealed the presence of two large pus tubes, one measuring six inches long, by three and a half inches thick, the other five inches long and three inches thick, bound by cord-like ligamentous adhesions, both fimbriæ were occluded, both ovaries contained in both masses, the left tube and ovary being attached to the sigmoid. Each mass was removed through an incision three inches long, being drawn out by their long axis. Each tube was examined microscopically. No bacteria and no gonococci found. There was an imperforate hymen and no history of exposure to infection.

This appears as rather a rare case. The patient left the hospital in twenty-one days. She was seen two weeks ago and has gained in weight, feels well, and has had no trouble since the operation.

## Figures and Tables

**Figure f1:**